# Supplementary material for: Stalled translation by mitochondrial stress upregulates a CNOT4-ZNF598 ribosomal quality control pathway important for tissue homeostasis
Source: Nat Commun. 2024 Feb 22;15:1637. doi: 10.1038/s41467-024-45525-3 (PMC10883933; doi:10.1038/s41467-024-45525-3)
Supplement: Supplementary file 1 — Supplementary Information [file 41467_2024_45525_MOESM1_ESM.pdf]

Supplementary Information

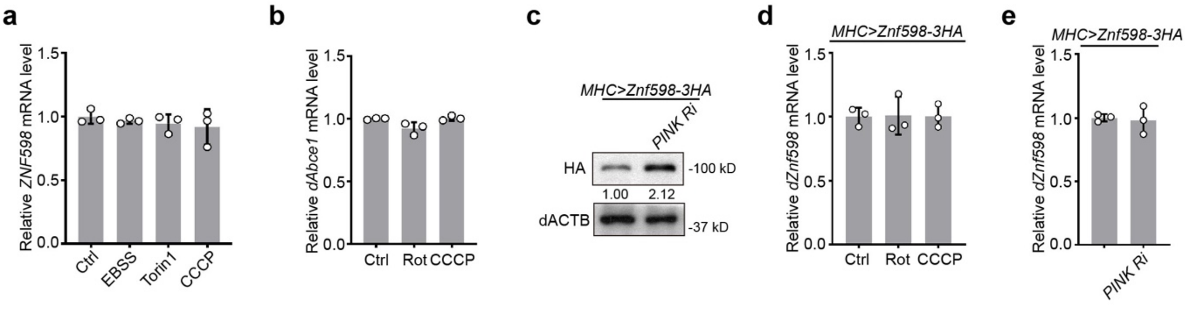

Fig. S1: Analysis of *ZNF598* mRNA level after mitochondrial damage in mammalian cells and *ZNF598* mRNA and protein expression in *PINK1* RNAi flies.

a RT-qPCR was used to assess *ZNF598* mRNA levels in EBSS-, Torin1- and CCCP-treated Hela cells. **b** RT-qPCR was used to assess *dABCE1* mRNA levels in rotenone- and CCCP-treated ABCE1 overexpressing flies. **c** Western blot analysis of HA-tagged ZNF598 transgene expression in *PINK1* RNAi fly muscle. **d** RT-qPCR was used to assess d*ZNF598* mRNA level in rotenone- and CCCP-treated ZNF598 overexpressing flies. **e** RT-qPCR was used to assess d*ZNF598* mRNA level in ZNF598-overexpressing *PINK1* RNAi flies.

Data are representative of at least 3 biologically independent experiments (mean±SD), n =3. Representative blot is from at least three independent experiments with similar results. Source data are provided as a Source Data file.

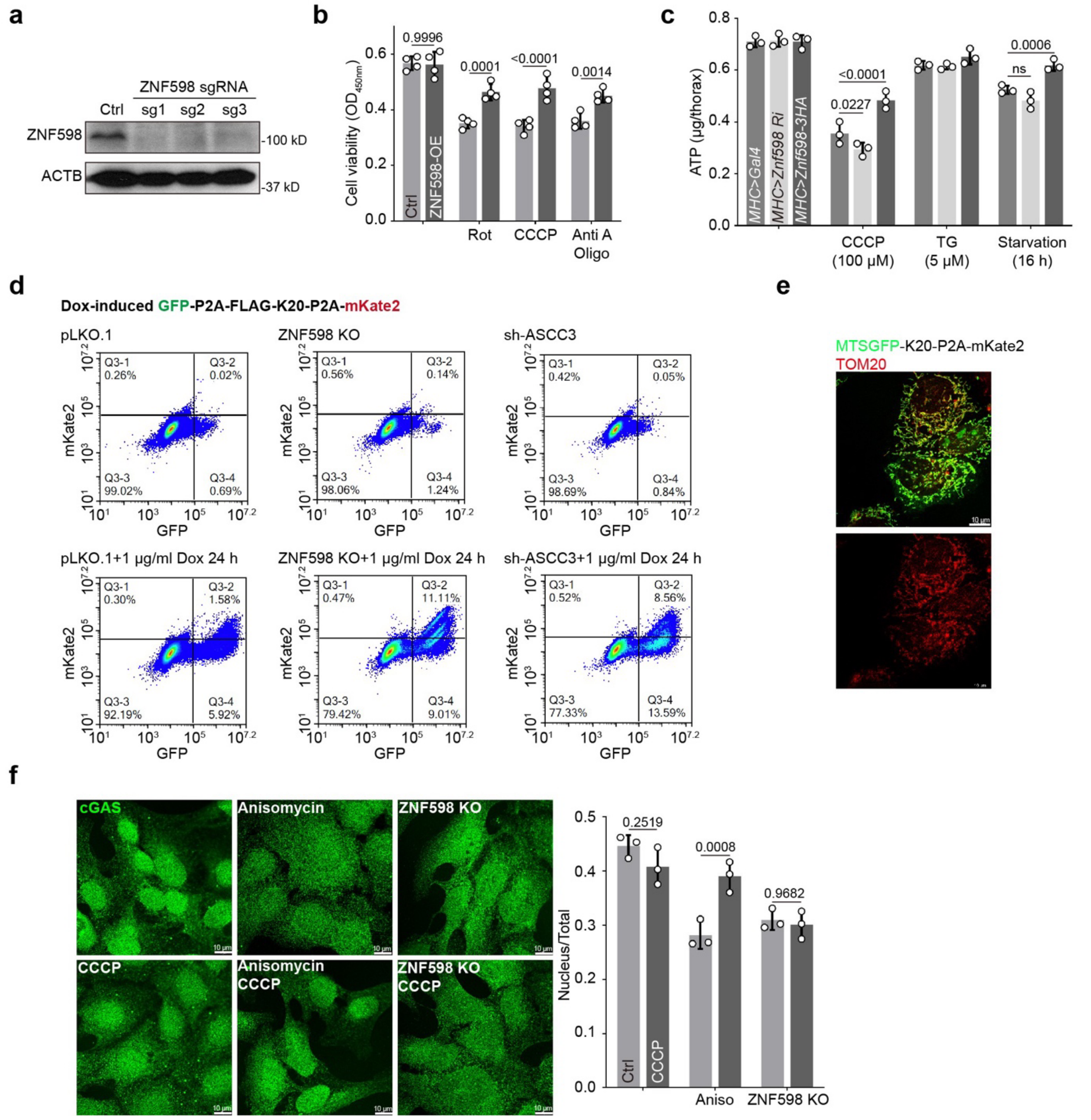

Fig. S2: Analysis of cellular phenotypes of ZNF598 deficient cells or ZNF598 deficient and OE flies.

a Immunoblots showing knockdown efficiency in CRISPR/Cas9-engineered ZNF598 knockout HEK293T cells. **b** Cell viability measurement using CCK8 assay in HEK293T cells stable overexpressing ZNF598 followed by treatment with rotenone, CCCP, and Antimycin A + Oligomycin. **c** ATP measurement in control, ZNF598 OE, or ZNF598 RNAi flies after CCCP,

TG, and starvation treatments for 16 h. **d** Flow cytometry image of GFP and RFP in normal, ZNF598 KO, and ASCC3 KD HEK293T cells expressing Dox-inducible GFP-P2A-Flag-K20-P2A-mKate2 after 1 μg/ml Dox incubation for 24 h. **e** Immunostaining of GFP and TOM20 in U2OS cells transfected with MTSGFP-K20-P2A-mKate2 for 24 h. **f** Immunostaining showing effect of anisomycin treatment or ZNF598 KO on cGAS subcellular distribution in U2OS cells with or without CCCP treatment. Bar graph shows data quantification.

Data are representative of at least 3 biologically independent experiments (mean±SD), n = 4 in **b**, n = 3 in **c**, **f**. Representative blot or image is from at least three independent experiments with similar results. p-values in **b** and **f** were calculated by two-way ANOVA (Sidak's test). p-values in **c** were calculated by two-way ANOVA (Tukey's test). ns = no significance. Source data are provided as a Source Data file.

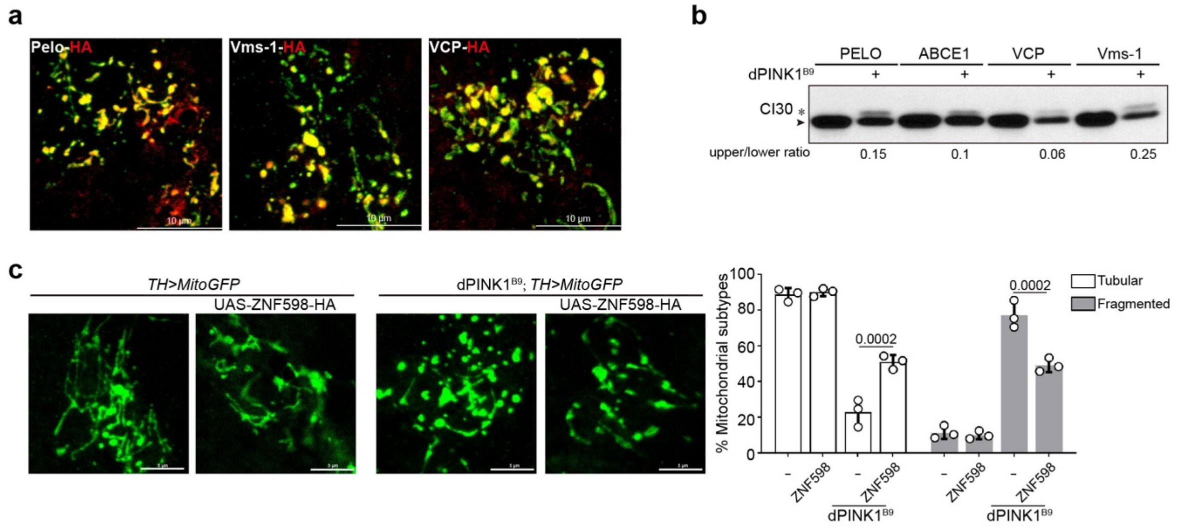

Fig. S3: Mitochondrial localization of RQC factors and effect of ZNF598 and other RQC factors on C-I30-u formation and mitochondrial morphology in *PINK1* mutant flies.

a Localization of HA-tagged RQC factors Pelo, Vms-1, and VCP to mitochondria of adult fly DA neurons. DA neuron mitochondria are marked with TH-Gal4-driven mito-GFP expression. **b** Western blot analysis showing effect of PELO, ABCE1, VCP, and VMS-1 OE on CAT-tailed C-I30-u formation in *PINK1*^{B9} fly muscle. **c** Effect of ZNF598 OE on mitochondrial morphology in DA neurons in the PPL1 cluster of adult *PINK1*^{B9} mutant fly brains. Bar graph shows quantification of relative proportions of tubular vs. fragmented mitochondria.

Data are representative of at least 3 biologically independent experiments (mean±SD), n =3. Representative blot or image is from at least three independent experiments with similar results. p-values were calculated by two-way ANOVA (Sidak's test). Source data are provided as a Source Data file.

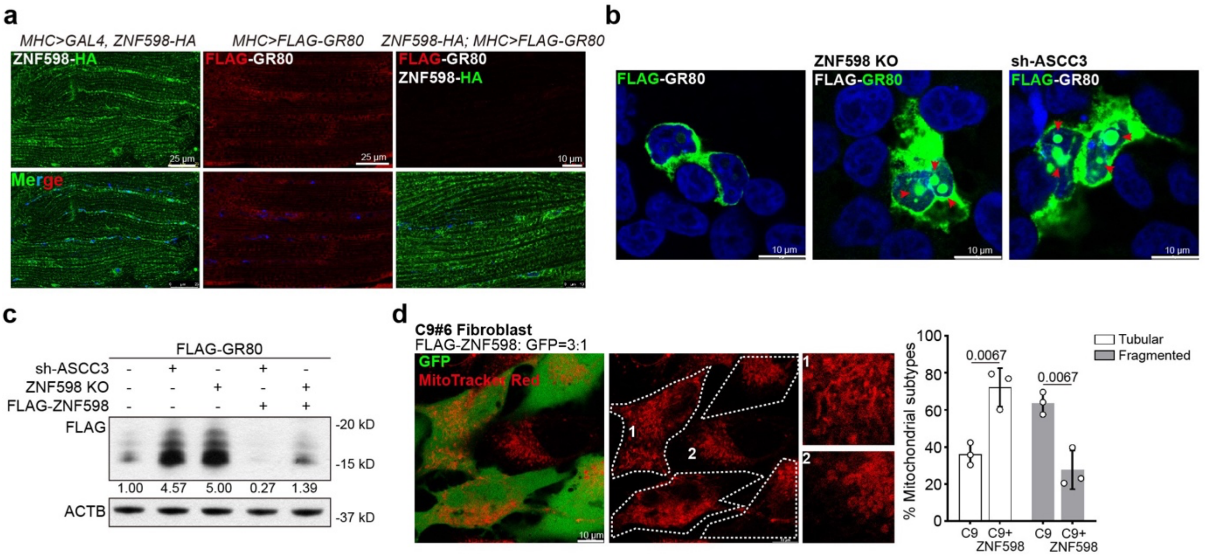

Fig. S4: ZNF598 regulates the quality control of stalled translation of C9ALS-associated poly(GR) and rescues poly(GR)-induced toxicity in *Drosophila* model and patient cells.

a Immunofluorescence staining for ZNF598 and FLAG-GR80 showing that ZNF598 decreases GR80 protein level in *Mhc>GR80* flies. **b** Immunofluorescence staining for FLAG-GR80 in ZNF598- or ASCC3-deficient HeLa cells. Arrowheads mark GR80 aggregates enriched in the nuclei of ZNF598- or ASCC3-deficient cells. **c** Immunoblots showing effect of ZNF598 OE on Flag-GR80 protein level in ZNF598- or ASCC3-deficient HeLa cells. **d** Mitotracker-Red staining of ZNF598 transfected and non-transfected C9ALS patient fibroblasts. Magnified views of cells 1 and 2 are shown to the right. Bar graph shows quantification of relative proportions of tubular vs. fragmented mitochondria.

Data are representative of at least 3 biologically independent experiments (mean±SD), n = 3. Representative blot or image is from at least three independent experiments with similar results. p-values were calculated by unpaired two-tailed *t*-test. Source data are provided as a Source Data file.

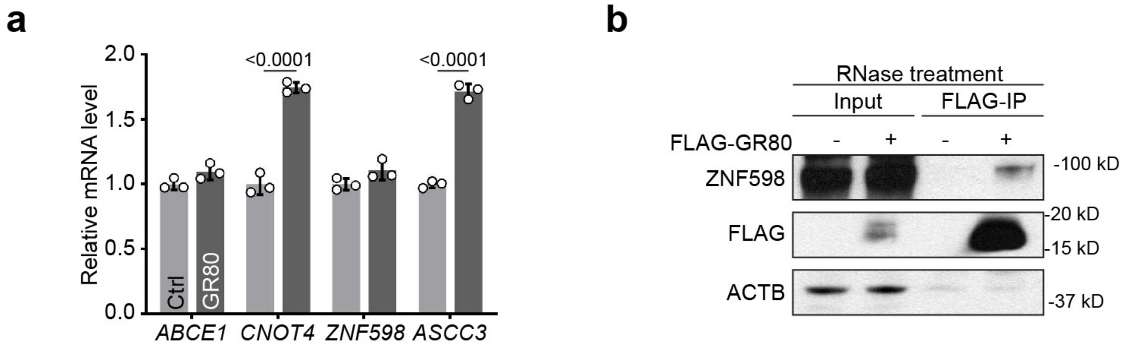

Fig. S5: RT-PCR analysis of RQC factor expression and poly(GR) interaction with ZNF598.

a RT-qPCR was used to assess *ABCE1*, *CNOT4*, *ZNF598*, and *ASCC3* mRNA levels in GR80 overexpressing HEK293T cells. **b** Immunoprecipitation analysis of the interaction between FLAG-GR80 and ZNF598 in HEK293T cell lysates with RNase treatment.

Data are representative of at least 3 biologically independent experiments (mean± SD), n = 3. Representative blot is from three independent experiments with similar results. p-values were calculated by unpaired two-tailed *t*-test. Source data are provided as a Source Data file.

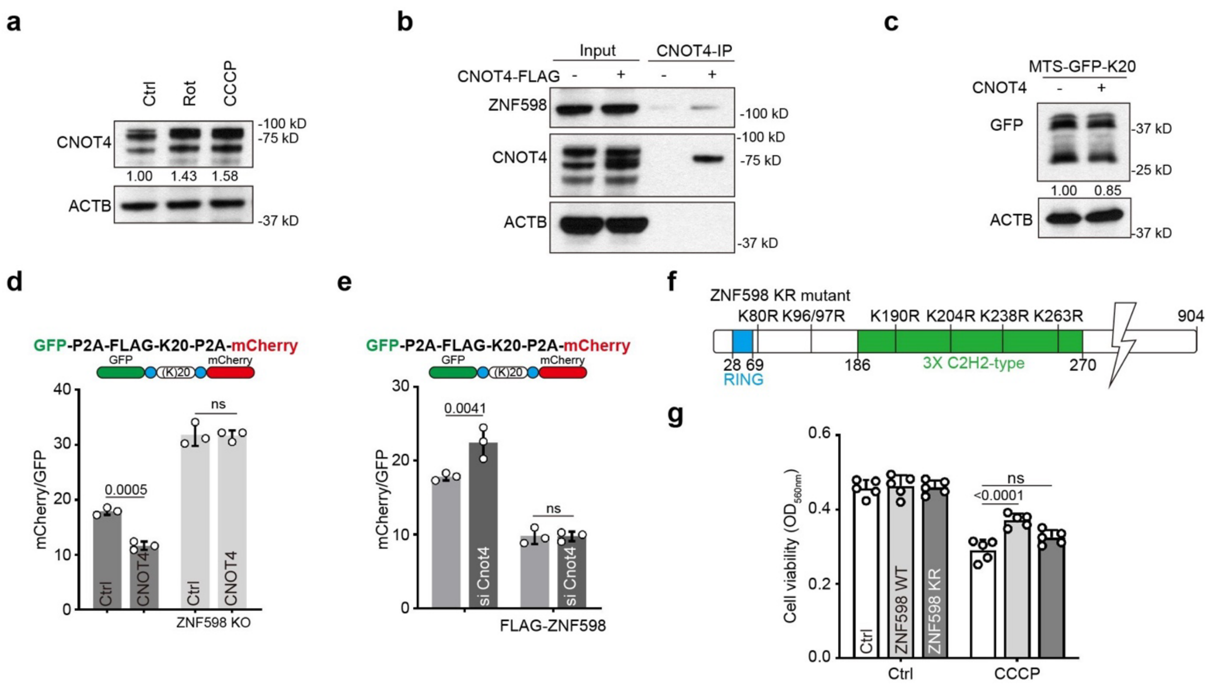

Fig. S6: CNOT4 is upregulated by mitochondrial stress and interacts with ZNF598.

a Immunoblots showing the level of endogenous CNOT4 in HEK293T cells treated with 2 μM Rotenone or 10 μM CCCP for 24 h. **b** Immunoprecipitation analysis of the interaction between endogenous ZNF598 and CNOT4-FLAG in transfected HEK293T cells. **c** Immunoblots showing effect of CNOT4 OE on MTS-GFP-K20 reporter expression. **d** The ratio of mCherry to GFP detected by flow cytometry showing effect of CNOT4 on GFP-FLAG-K20-mCherry reporter expression in ZNF598 KO HEK293T cells. **e** The ratio of mCherry to GFP detected by flow cytometry showing effect of ZNF598 on GFP-FLAG-K20-mCherry reporter expression in CNOT4 KD HEK293T cells. **f** Schematic of ZNF598-KR mutant. **g** Cell viability measurement using CCK8 assay in cells transfected with ZNF598 WT or ZNF598 KR. Cells were treated with 20 μM CCCP (24 h).

Data are representative of at least 3 biologically independent experiments (mean± SD), n = 3. Representative blot is from three independent experiments with similar results. p-values in **d**

and **e** were calculated by two-way ANOVA (Sidak's test). p-values in **g** were calculated by two-way ANOVA (Tukey's test). ns = no significance. Source data are provided as a Source Data file.

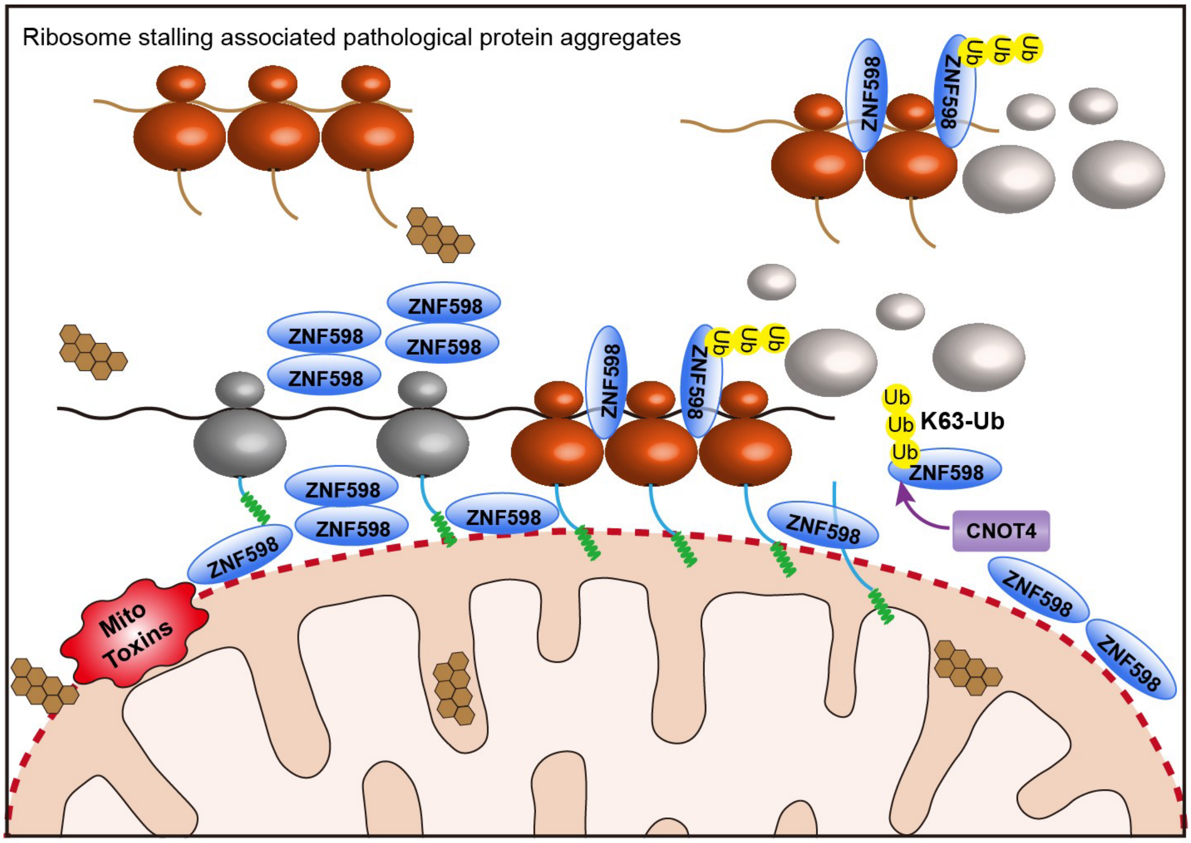

Fig. S7: A schematic diagram of mitochondrial translocation and CNOT4-mediated ubiquitination of ZNF598 during ribosome-associated translational quality control in response to mitochondrial stress.

When mitochondria are damaged, mitochondrial outer membrane associated ribosomes translating mRNAs encoding mitonuclear proteins (e.g. C-I30) are stalled. In response, ZNF598 is recruited to mitochondrial outer membrane to handle stalled ribosomes; in the process, CNOT4 catalyzes K63-linked regulatory ubiquitination of ZNF598, helping to relieve stalled ribosomes and reduce pathological protein aggregates caused by ribosome stalling.
