# Supplementary material for: Stalled translation by mitochondrial stress upregulates a CNOT4-ZNF598 ribosomal quality control pathway important for tissue homeostasis
Source: Nat Commun. 2024 Feb 22;15:1637. doi: 10.1038/s41467-024-45525-3 (PMC10883933; doi:10.1038/s41467-024-45525-3)
Supplement: Supplementary file 3 — Reporting Summary [file 41467_2024_45525_MOESM3_ESM.pdf]

nature portfolio

Corresponding author(s): Ji Geng; Binwgei Lu

Last updated by author(s): 01/05/2024

Reporting Summary

Nature Portfolio wishes to improve the reproducibility of the work that we publish. This form provides structure for consistency and transparency in reporting. For further information on Nature Portfolio policies, see our Editorial Policies and the Editorial Policy Checklist.

Statistics

For all statistical analyses, confirm that the following items are present in the figure legend, table legend, main text, or Methods section.

n/a	Confirmed	
☐	☒	The exact sample size (*n*) for each experimental group/condition, given as a discrete number and unit of measurement
☐	☒	A statement on whether measurements were taken from distinct samples or whether the same sample was measured repeatedly
☐	☒	The statistical test(s) used AND whether they are one- or two-sided<br>*Only common tests should be described solely by name; describe more complex techniques in the Methods section.*
☒	☐	A description of all covariates tested
☐	☒	A description of any assumptions or corrections, such as tests of normality and adjustment for multiple comparisons
☐	☒	A full description of the statistical parameters including central tendency (e.g. means) or other basic estimates (e.g. regression coefficient) AND variation (e.g. standard deviation) or associated estimates of uncertainty (e.g. confidence intervals)
☐	☒	For null hypothesis testing, the test statistic (e.g. F, t, r) with confidence intervals, effect sizes, degrees of freedom and P value noted<br>*Give P values as exact values whenever suitable.*
☒	☐	For Bayesian analysis, information on the choice of priors and Markov chain Monte Carlo settings
☒	☐	For hierarchical and complex designs, identification of the appropriate level for tests and full reporting of outcomes
☒	☐	Estimates of effect sizes (e.g. Cohen's *d*, Pearson's *r*), indicating how they were calculated

Our web collection on statistics for biologists contains articles on many of the points above.

Software and code

Policy information about availability of computer code

Data collection	Leica Application Suite X (LAS X) software for imaging collection.
Data analysis	Fiji/Image J for WB analysis. All statistical analyses were performed with GraphPad Prism Software Version 9.0

For manuscripts utilizing custom algorithms or software that are central to the research but not yet described in published literature, software must be made available to editors and reviewers. We strongly encourage code deposition in a community repository (e.g. GitHub). See the Nature Portfolio guidelines for submitting code & software for further information.

Data

Policy information about availability of data

All manuscripts must include a data availability statement. This statement should provide the following information, where applicable:

- Accession codes, unique identifiers, or web links for publicly available datasets
- A description of any restrictions on data availability
- For clinical datasets or third party data, please ensure that the statement adheres to our policy

All data supporting the findings of this study including the uncropped western blot (WB) scans are available within the paper and its Supplementary Information.

Research involving human participants, their data, or biological material

Policy information about studies with human participants or human data. See also policy information about sex, gender (identity/presentation), and sexual orientation and race, ethnicity and racism.

Reporting on sex and gender	N/A
Reporting on race, ethnicity, or other socially relevant groupings	N/A
Population characteristics	N/A
Recruitment	N/A
Ethics oversight	N/A

Note that full information on the approval of the study protocol must also be provided in the manuscript.

Field-specific reporting

Please select the one below that is the best fit for your research. If you are not sure, read the appropriate sections before making your selection.

☒ Life sciences ☐ Behavioural & social sciences ☐ Ecological, evolutionary & environmental sciences

For a reference copy of the document with all sections, see nature.com/documents/nr-reporting-summary-flat.pdf

Life sciences study design

All studies must disclose on these points even when the disclosure is negative.

Sample size	No sample size calculation was performed. We performed a minimum of 3 biological replicates. For drosophila experiments, due to the limitation of genotype, the fly number is over 10 flies in each experiment.
Data exclusions	No data was excluded for data analyses, unless we noticed there were traceable operation errors in the experiments
Replication	Immunoblots, Drosophila immunofluorescence staining shown were replicated 3 times independently unless otherwise stated in figure legends. And all attempts at replication were successful.
Randomization	Mice and drosophila were randomly assigned to different treatment groups. For in vitro experiments, all samples were completely randomized to treatment groups.
Blinding	The investigators were single blinding when collecting and analyzing the data.

Reporting for specific materials, systems and methods

We require information from authors about some types of materials, experimental systems and methods used in many studies. Here, indicate whether each material, system or method listed is relevant to your study. If you are not sure if a list item applies to your research, read the appropriate section before selecting a response.

Materials & experimental systems

n/a	Involved in the study	
☐	☒	Antibodies
☐	☒	Eukaryotic cell lines
☒	☐	Palaeontology and archaeology
☐	☒	Animals and other organisms
☒	☐	Clinical data
☒	☐	Dual use research of concern
☒	☐	Plants

Methods

n/a	Involved in the study	
☒	☐	ChIP-seq
☐	☒	Flow cytometry
☒	☐	MRI-based neuroimaging

Antibodies

Antibodies used	Anti-ZNF598 antibody (HPA041760, Sigma Aldrich) (1:1000)<br>Anti-β-Actin antibody (sc-47778, Santa Cruz) (1:5000)

Anti-RACK1 antibody (sc-17754, Santa Cruz) (1:13000)
Anti-HA antibody (51064-2-AP, Proteintech) (1:3000)
Anti-RPS6 antibody (#2217, Cell signaling tehnology) (1:1000)
Anti-ANKZF1 antibody (20447-1-AP, Proteintech) (1:1000)
Anti-TOM20 antibody (sc-17764, Santa Cruz) (1:1000)
Anti-ASCC3 antibody (A304-014A, Bethyl lab) (1:500)
Anti-GFP antibody (66002-1-Ig, Proteintech) (1:3000)
Anti-FLAG antibody (F1804, Sigma Aldrich) (1:3000)
Anti-RFP antibody (PA1-986, Invitrogen) (1:1000)
Anti-VDAC1 (MABN504, Millipore) (1:3000)
Anti-CHCHD3 (25625-1-AP, Proteintech) (1:1000)
Anti-NDUFS3 antibody [17D95] (ab14711, Abcam) (1:1000)
Anti-dTH (Lab Stock) (1:1000)
Anti-GR repeats antibody (23978-1-AP, Proteintech) (1:1000)
Anti-FMRP antibody (13755-1-AP, Proteintech) (1:1000)
Anti-RPS3 antibody (#A303-841A, Bethyl Lab) (1:5000)
Anti-CNOT4 antibody (67798-1-Ig, Proteintech) (1:1000)
Anti-RPS10 antibody (ab151550, Abcam) (1:5000)
Anti-EDF1 antibody (A2283, Abclonal) (1:1000)
Anti-ABCE1 antibody (28548-1-AP, Proteintech) (1:1000)
Anti-PELO antibody (10582-1-AP, Proteintech) (1:1000)
Anti-LTN1 antibody (28452-1-AP, Proteintech) (1:1000)
Anti-4EHP antibody (12227-1-AP, Proteintech) (1:1000)
Anti-GIGYF2 antibody (24790-1-AP, Proteintech) (1:1000)
Anti-Calmodulin antibody (#35944, Cell signaling technology) (1:1000)
Anti-mCherry antibody (#26765-1-AP, Proteintech) (1:3000)
Anti-rabbit IgG, HRP-linked Antibody (#7074, Cell signaling Technology) (1:10000)
Anti-mouse IgG, HRP-linked Antibody (#7076, Cell signaling Technology) (1:10000)
Goat anti-Mouse IgG, IgM (H+L) Secondary Antibody, Alexa Fluor™ 488 (# A-10680) (1:1000)
Goat anti-Mouse IgG (H+L) Cross-Adsorbed Secondary Antibody, Alexa Fluor™ 555 (# A-21422) (1:1000)
Goat anti-Rabbit IgG (H+L) Cross-Adsorbed Secondary Antibody, Alexa Fluor™ 488 (# A-11008) (1:1000)
Goat anti-Rabbit IgG (H+L) Cross-Adsorbed Secondary Antibody, Alexa Fluor™ 568 (# A-11011) (1:1000)
Goat anti-Rabbit IgG (H+L) Cross-Adsorbed Secondary Antibody, Alexa Fluor™ 633 (# A-21070) (1:1000)

Validation

All the antibodies are commercially available and validated by manufacturers. The indicated species and application can be accessed on the manufacturers' website. These antibodies validation have been confirmed on human cell lysates and fly lysates using western blot or immunofluorescence according to the product introductions. The specific product slinks were as following:
Anti-ZNF598 antibody (https://www.sigmaaldrich.com/US/en/product/sigma/hpa041760)
Anti-β-Actin antibody (https://www.scbt.com/p/beta-actin-antibody-c4)
Anti-RACK1 antibody (https://www.scbt.com/p/rack1-antibody-b-3)
Anti-HA antibody (https://www.ptglab.com/products/HA-tag-Antibody-51064-2-AP.htm)
Anti-RPS6 antibody (https://www.cellsignal.com/products/primary-antibodies/s6-ribosomal-protein-5g10-rabbit-mab/2217)
Anti-ANKZF1 antibody (https://www.ptglab.com/products/ANKZF1-Antibody-20447-1-AP.htm)
Anti-TOM20 antibody (https://www.scbt.com/p/tom20-antibody-f-10)
Anti-ASCC3 antibody (https://www.fortislife.com/products/primary-antibodies/rabbit-anti-ascc3-antibody/BETHYL-A304-014)
Anti-GFP antibody (https://www.ptgcn.com/products/eGFP-Antibody-66002-1-Ig.htm)
Anti-FLAG antibody (https://www.sigmaaldrich.com/US/en/product/sigma/f1804)
Anti-RFP antibody (https://www.thermofisher.cn/cn/zh/antibody/product/RFP-Antibody-Polyclonal/PA1-986)
Anti-VDAC1 (https://www.emdmillipore.com/US/en/product/Anti-VDAC1-Antibody-clone-N152B-23,MM_NF-MABN504?ReferrerURL=https%3A%2F%2Fwww.google.com%2F&bd=1)
Anti-CHCHD3 (https://www.ptgcn.com/products/CHCHD3-Antibody-25625-1-AP.htm)
Anti-NDUFS3 antibody [17D95] (https://www.abcam.com/products/primary-antibodies/ndufs3-antibody-17d95-ab14711.html)
Anti-dTH (Lab Stock)
Anti-GR repeats antibody (https://www.ptglab.com/products/GR-repeat-Antibody-23978-1-AP.htm)
Anti-FMRP antibody (https://www.ptgcn.com/products/FMR1-Antibody-13755-1-AP.htm)
Anti-RPS3 antibody (https://www.fortislife.com/products/primary-antibodies/rabbit-anti-rps3-antibody/BETHYL-A303-841)
Anti-CNOT4 antibody (https://www.ptglab.com/products/CNOT4-Antibody-67798-1-Ig.htm)
Anti-RPS10 antibody (https://www.abcam.com/rps10-antibody-epr8545-ab151550.html)
Anti-EDF1 antibody (https://abclonal.com/catalog-antibodies/KOValidatedEDF1RabbitpAb/A2283)
Anti-ABCE1 antibody (https://www.ptglab.com/products/ABCE1-Antibody-28548-1-AP.htm)
Anti-PELO antibody (https://www.ptgcn.com/products/PELO-Antibody-10582-1-AP.htm)
Anti-LTN1 antibody (https://www.ptglab.com/products/RNF160-Antibody-28452-1-AP.htm)
Anti-4EHP antibody (https://www.ptglab.com/products/EIF4E2-Antibody-12227-1-AP.htm)
Anti-GIGYF2 antibody (https://www.ptgcn.com/products/GIGYF2-Antibody-24790-1-AP.htm)
Anti-Calmodulin antibody (https://www.cellsignal.com/products/primary-antibodies/calmodulin-d1f7j-rabbit-mab/35944)
Anti-mCherry antibody (https://www.ptglab.com/products/mCherry-Antibody-26765-1-AP.htm)
Anti-rabbit IgG, HRP-linked Antibody (https://www.cellsignal.com/products/secondary-antibodies/anti-rabbit-igg-hrp-linked-antibody/7074)
Anti-mouse IgG, HRP-linked Antibody (https://www.cellsignal.com/products/secondary-antibodies/anti-mouse-igg-hrp-linked-antibody/7076)
Goat anti-Mouse IgG, IgM (H+L) Secondary Antibody, Alexa Fluor™ 488 (https://www.thermofisher.com/antibody/product/Goat-anti-Mouse-IgG-IgM-H-L-Secondary-Antibody-Polyclonal/A-10680)
Goat anti-Mouse IgG (H+L) Cross-Adsorbed Secondary Antibody, Alexa Fluor™ 555 (https://www.thermofisher.com/antibody/product/Goat-anti-Mouse-IgG-H-L-Cross-Adsorbed-Secondary-Antibody-Polyclonal/A-21422)
Goat anti-Rabbit IgG (H+L) Cross-Adsorbed Secondary Antibody, Alexa Fluor™ 488 (https://www.thermofisher.com/antibody/product/Goat-anti-Rabbit-IgG-H-L-Cross-Adsorbed-Secondary-Antibody-Polyclonal/A-11008)

Goat anti-Rabbit IgG (H+L) Cross-Adsorbed Secondary Antibody, Alexa Fluor™ 568 (https://www.thermofisher.com/antibody/product/Goat-anti-Rabbit-IgG-H-L-Cross-Adsorbed-Secondary-Antibody-Polyclonal/A-11011)
Goat anti-Rabbit IgG (H+L) Cross-Adsorbed Secondary Antibody, Alexa Fluor™ 633 (https://www.thermofisher.com/antibody/product/Goat-anti-Rabbit-IgG-H-L-Cross-Adsorbed-Secondary-Antibody-Polyclonal/A-21070)

Eukaryotic cell lines

Policy information about cell lines and Sex and Gender in Research

Cell line source(s)	U2OS cells (Gift from the Kopito Lab, Stanford University)<br>HEK293T cells (CRL-3216 - ATCC)<br>HELA cell (Lab stock)<br>ALS-C9 fibroblasts (Gift from the Gitler lab, Stanford University)
Authentication	None of the cell lines used were detected by STR analysis.
Mycoplasma contamination	Cell lines were not tested for mycoplasma contamination. In addition, Plasmocin™ prophylactic was added in our medium to avoid mycoplasma contamination.
Commonly misidentified lines (See ICLAC register)	No commonly misidentified cell lines were used in the study

Animals and other research organisms

Policy information about studies involving animals; ARRIVE guidelines recommended for reporting animal research, and Sex and Gender in Research

Laboratory animals	C57BL/6 mice (male, 10-12 weeks old, 23-25 g) were purchased from Jackson Laboratory. The animals were housed, five per cage with food and water ad libitum, on a 12 h light/dark cycle with lights on at 06:00 h and controlled (22-23°C) temperature. Animal welfare and experimental procedures were carried out strictly in accordance with the related ethical regulations of Stanford University (Protocol No. 22400).<br>Drosophila culture and crosses were performed according to standard procedures. Adult flies were generally raised at 25°C and with 12/12 hr dark/light cycles. Male flies with one month old were used in the experiment.
Wild animals	No wild animal
Reporting on sex	Male
Field-collected samples	the study did not include field-collected samples.
Ethics oversight	Animal welfare and experimental procedures were carried out strictly in accordance with the related ethical regulations of Stanford University (Protocol No. 22400).

Note that full information on the approval of the study protocol must also be provided in the manuscript.

Flow Cytometry

Plots

Confirm that:

☒ The axis labels state the marker and fluorochrome used (e.g. CD4-FITC).

☒ The axis scales are clearly visible. Include numbers along axes only for bottom left plot of group (a 'group' is an analysis of identical markers).

☒ All plots are contour plots with outliers or pseudocolor plots.

☒ A numerical value for number of cells or percentage (with statistics) is provided.

Methodology

Sample preparation	*Describe the sample preparation, detailing the biological source of the cells and any tissue processing steps used.*
Instrument	*Identify the instrument used for data collection, specifying make and model number.*
Software	*Describe the software used to collect and analyze the flow cytometry data. For custom code that has been deposited into a community repository, provide accession details.*
Cell population abundance	*Describe the abundance of the relevant cell populations within post-sort fractions, providing details on the purity of the samples and how it was determined.*

Gating strategy

Describe the gating strategy used for all relevant experiments, specifying the preliminary FSC/SSC gates of the starting cell population, indicating where boundaries between "positive" and "negative" staining cell populations are defined.

☐ Tick this box to confirm that a figure exemplifying the gating strategy is provided in the Supplementary Information.
